# African forest elephant movements depend on time scale and individual behavior

**DOI:** 10.1038/s41598-021-91627-z

**Published:** 2021-06-16

**Authors:** Christopher Beirne, Thomas M. Houslay, Peter Morkel, Connie J. Clark, Mike Fay, Joseph Okouyi, Lee J. T. White, John R. Poulsen

**Affiliations:** 1 Nicholas School of the Environment, Duke University, PO Box 90328, Durham, 27708 NC USA; 2 Department of Zoology, University of Cambridge, Cambridge, CB2 fJ UK; 3 Independent Researcher, Karasburg, Namibia; 4 Agence Nationale Des Parcs Nationaux, Batterie IV, BP. 20379, Libreville, Gabon; 5 Institut de Recherche en Ecologie Tropicale, BP. 13354, Libreville, Gabon; 6 African Forest Ecology Group, School of Natural Sciences, University of Stirling, Stirling, UK

**Keywords:** Ecology, Animal migration, Behavioural ecology, Conservation biology, Tropical ecology, Animal behaviour

## Abstract

The critically endangered African forest elephant (*Loxodonta cyclotis*) plays a vital role in maintaining the structure and composition of Afrotropical forests, but basic information is lacking regarding the drivers of elephant movement and behavior at landscape scales. We use GPS location data from 96 individuals throughout Gabon to determine how five movement behaviors vary at different scales, how they are influenced by anthropogenic and environmental covariates, and to assess evidence for behavioral syndromes—elephants which share suites of similar movement traits. Elephants show some evidence of behavioral syndromes along an ‘idler’ to ‘explorer’ axis—individuals that move more have larger home ranges and engage in more ‘exploratory’ movements**.** However, within these groups, forest elephants express remarkable inter-individual variation in movement behaviours. This variation highlights that no two elephants are the same and creates challenges for practitioners aiming to design conservation initiatives.

## Introduction

Movement is a fundamental characteristic of animal biology, and the movement decisions animals make have profound implications across individual, population and ecosystem-levels. At the individual-level, movement behaviour influences fitness through the ability to find resources, survive and reproduce^1,2^. At the population-level, movement characteristics can influence interactions with competitors, predators and disease^3,4^. At the ecosystem-level, animal movement can determine the location of dispersed pollen, seeds and nutrients and the spatial intensity of herbivory, predation and disturbance^5^. Conserving species and functioning ecosystems, therefore, depends on understanding how and why animals move and the consequences of such decisions.

There is increasing recognition of the importance in quantifying the role of, and variation associated with, individual movement patterns. This change is assisted by the increasing availability of high quality animal tracking data and the associated development of powerful analytical frameworks which can quantify movement behavior at sufficiently fine temporal scales^1^*.* Where explored, individual-level variation in movement behaviors is often a typical feature of movement data sets^6–8^ and can translate into individual variation in resource acquisition, body mass, reproductive output, and survival^9^. Individual variation can facilitate coexistence of multiple individuals through reductions in competition for resources^10,11^ and has profound implications for management of human-wildlife conflict through identification of problem individuals^12^. Characterizing multiple movement behaviors at the individual-level can also reveal behavioral syndromes—correlated suites of behaviors that can constrain or enhance how individuals and populations respond to anthropogenic disturbance or novel environments^13^*.* As GPS tracking typically involves following multiple individuals across ecologically meaningful time periods, it is perfectly suited for assessments of behavioral syndromes. To date, studies with a sufficient number of tracked animals over a large enough spatial area to assess heterogeneity in movements with environmental and anthropogenic factors are rare, even in large mammals^13^, but see^6,7,14^. Ultimately, identification of factors governing animal movement and behavioral syndromes could lead to targeted regional- or syndrome-specific management recommendations, resulting in more effective conservation and resolution of human-wildlife conflict.

The African forest elephant (*Loxodonta cyclotis*) plays a pivotal ecological role in seed dispersal, nutrient recycling and herbivory, ultimately influencing the structure and functioning of Afro-tropical forests^15^. However, research into forest elephant movement behavior has been limited to small sample sizes and small temporal and spatial scales, as direct observations of forest elephants are largely restricted to non-forest, open habitats that compose a small fraction of their extant range. Given the rapid rate of decline of forest elephant populations across their range (60–80% within ten years:^16,17^), landscape-scale characterization of the drivers of movement, and its associated individual variation, is vital for the design of effective conservation and management strategies^18^, forecasting how forest elephants will cope with future environmental change^18,19^, and determining how their absence will transform tropical forest ecosystems^15^.

We derive five individual-level movement behaviors from forest elephant GPS tracking data: movement distance, home range size, site fidelity, diurnality and exploratory behavior—each of which captures an important aspect of movement ecology. ‘Movement distance’ represents the sum of all movements across a temporal period of interest, reflects individual activity levels and energy use, and is influenced by social interactions, such as mating and competition, and forage quality and distribution^20^. ‘Home range size’ reflects the amount of space that forest elephants require to satisfy their dietary, social and reproductive requirements in a given time step^21^. Home range is an important parameter for designing wildlife management strategies—how much area does a forest elephant require in a given period of time? ‘Site fidelity’ reflects the degree to which individuals remain in one location or move freely around the landscape and has been shown to govern survival and reproductive success in multiple taxa^22,23^. Temporal variation in site fidelity of large herbivores can arise through temporal and spatial heterogeneity in forage quality or predation risk^23^ and is important for understanding human-elephant conflict. ‘Diurnality’ reflects the diel pattern of movement activity. Diurnal anthropogenic disturbance may cause individuals to become more nocturnal^24–28^. Finally, ‘exploratory movements’ are long, directionally persistent movements to new locations. The relative amount of time individuals spend in exploratory movements versus resting/foraging (shorter movements with low directional persistence) is important for understanding individual foraging decisions, social interactions and responses to anthropogenic disturbances^29,30^. Characterizing these movement behaviors and the factors that influence them is important for effective conservation; for example, to ensure that reserves or corridors are large enough, properly configured, and allow for seasonal and/or anthropogenically-induced behavioral changes.

The factors that influence how individuals move are varied and complex^31^, but can be broadly classified as either ‘intrinsic’ or ‘extrinsic’^32^. Intrinsic factors reflect the physiological state of the individual, such as sex or body condition. Sex may have a marked influence on forest elephant movement behaviors through sex-specific life history requirements, physiology or sexual-size dimorphism^33^. Forest elephants have manifested some sex-related differences in movement behavior^34^. Extrinsic factors are external environmental (e.g., habitat quality) and anthropogenic (e.g., poaching) pressures on individuals. In terms of environmental factors, savannah elephants track rainfall related changes in vegetation dynamics^35^ and forest elephants exhibit seasonal differences in habitat use driven by precipitation^34^. In terms of anthropogenic factors, forest elephants outside protected areas tend to have smaller home ranges than inside protected areas^36^ and typically avoid large roads and other human infrastructure^37–39^. The extent to which these factors influence movement characteristics is likely to vary with temporal scale^40^, as different drivers may operate at different temporal and spatial scales^41^. To date, the relative importance of intrinsic and extrinsic factors on forest elephant movement, and how it varies with temporal scale, remain largely unknown.

Here, we employ data from the largest forest elephant GPS collaring program to date to explore how the movement behaviors of 96 individuals in Gabon are influenced by both intrinsic (sex) and extrinsic factors (rainfall, temperature seasonality and anthropogenic disturbance). We evaluate five important movement attributes (movement distance, home range size, site fidelity, diurnality and exploratory behavior) across two temporal scales (annual and monthly). Specifically, we address three key questions: (i) What are the key drivers of elephant movement behavior? (ii) Does the relative importance of drivers vary depending on temporal scale? (iii) Is there evidence of repeatable inter-individual differences in behavior responses (inter-individual variation) and/or individual-level covariance between the different movement behaviors (behavioral syndromes)? We discuss the results in terms of how they influence our understanding of the ecological role forest elephants play in shaping Afrotropical forests, and how this information can be used to improve elephant management and conservation strategies.

## Methods

### Study area

Gabon is the second most forested country in the world, with 88% of its area (~ 267,000 km^2^) covered by tropical rain forest^42^ and the largest extant population of forest elephants^17^. The annual mean temperature of Gabon is about 25.0 °C (1901–2015). On average, temperatures are highest in March (26.2 °C) and lowest in July (23.1 °C). Average annual precipitation is about 1800 mm, with a dry season from June through August (mean = 25.4 mm month^−1^) and a bimodal wet season with peaks from March to May (mean = 203.3 mm month^−1^) and October to December (mean = 239.2 mm month^−1^) (http://sdwebx.worldbank.org/climateportal/index.cfm, verified on Jul. 18, 2017). Annual precipitation varies along a west-to-east gradient, declining from 2650 mm along the Atlantic coast to 1400 mm in the southeast of the country.

### Elephant GPS location data

**Figure 1:**
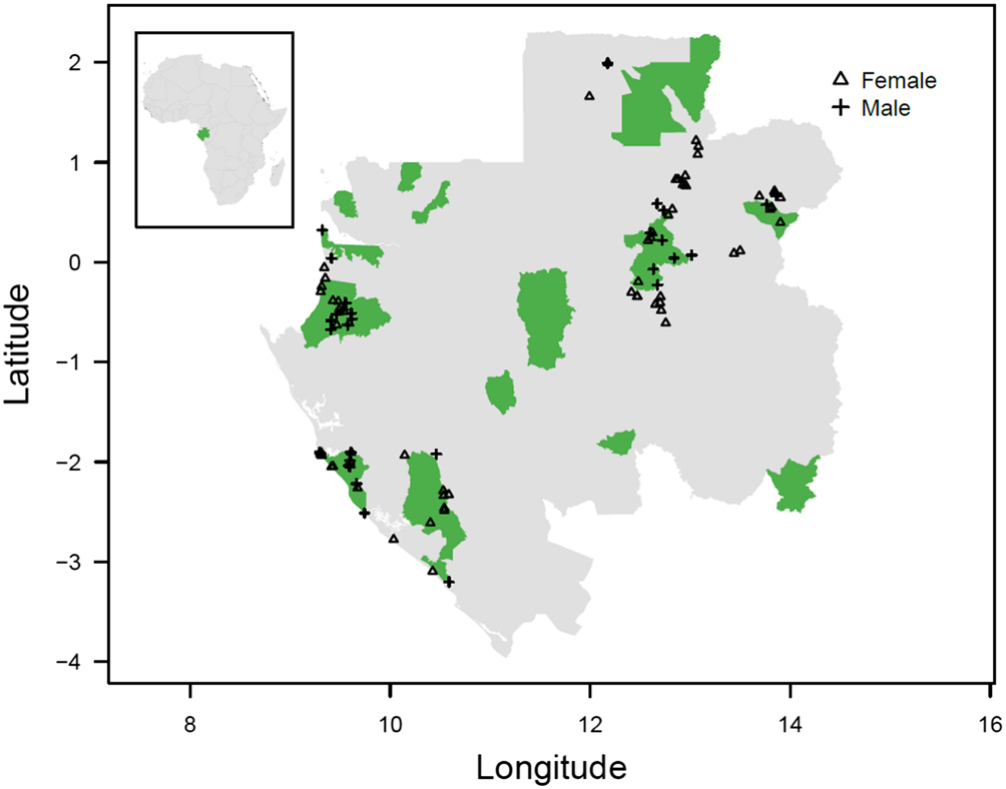
Map of the study area. The inset denotes the location of Gabon (green) within Africa; the main map shows mainland Gabon (grey); the distribution of protected areas (green); and the first GPS location of each elephant by sex in the dataset (black points; n = 96). The map was generated with R version 4.0.3 (https://www.r-project.org/).

The forest elephant collaring program commenced in October 2015 with the collaring of 12 elephants in the Wonga Wongué presidential reserve. An additional 84 elephants were collared during five collaring events: April 2016 (n = 22); January 2017 (n = 16); December 2017 (n = 24); March 2019 (n = 12); and October 2019 (n = 10) in and around five national parks (Ivindo, Loango, Moukalaba Doudou, Minkébé, and Mwagne; Fig. 1; Supplementary Table 1). The field team attempted to collar a balanced sample of males and females; however, males were scarce in some locations (e.g., Moukalaba Doudou). For a full description of capture and collaring protocols see^34^. All collars were set to 1-h GPS fix intervals. For a short time after the collars were deployed and after each collar had functioned for two years, a 4- or 12-h interval was used. To preclude the possibility that collar interval influenced the analyses below, we only used data with fix intervals of one hour. To remove possible fix errors, we pre-filtered the data, removing points that exceeded a threshold speed of 7 km/h in the ‘Save the Elephants’ application. The final dataset contains 1,219,344 GPS locations from 96 unique individuals. To ensure that results from statistical models were not driven by systematic variation in missing fixes, we verified that GPS locations were temporally regular between individuals and regions: 95.1% of fixes occurred within 2 h of the previous fix, and 99.0% occurred within 3 h with negligible between individual, sex and regional variation (Supplementary Fig. 1).

### Movement behaviors

For each elephant at each temporal scale, annual or monthly, we quantified the following behaviors: ‘home range’—the area of the 95% kernel density estimate using the ‘adeHabitatHR’ package^43^ with a fixed smoothing parameter, h, which represented the mean of all h parameters when it was allowed to vary at each time step for each temporal scale (annual = 1220; monthly = 920); ‘movement distance’—the sum of the Euclidian distance between all GPS fixes for each given temporal scale; ‘site fidelity’—the % overlap in home ranges between one time step (*t*) and the preceding time step (*t-1*); ‘diurnality’—following Hoogenboom et al. (1984), the sum of all movement distances during day time (06:00 to 18:00) minus the sum of the movement during night time (18:00 to 06:00) divided by the total movement distance (00:00 to 24:00) in each given day, averaged across the time step of interest (monthly or annual) (+ 1 = exclusively diurnal movement and − 1 = exclusively nocturnal movement); ‘exploratory behaviour’—the proportion of time spent in an exploratory state across the time step of interest. We used hidden Markov models in the package ‘momentuHMM’^29^ to classify each movement step as either ‘encamped’ (small movements with high turning angles—resting or foraging) or ‘exploratory’ (longer movements with directional persistence—movement between patches). Hidden Markov models use time series analysis to classify movement tracks into behavioral modes—they account for the high degree of autocorrelation in telemetry data and are robust when observational error is low and homogenous across individuals (as in this study: Supplementary Fig. 1). They have recently been used to elucidate spatial memory^44^, detect musth-related movement behavior^45^ and explore crop raiding decisions in savannah elephants^30^. We used a gamma distribution to describe the step lengths, a von Mises distribution to describe turning angles, and the Viterbi algorithm to estimate the most likely sequence of movement states to have generated the observations.

### Temporal scales

We estimated 100 annual home ranges, movement distances, activity ratios and exploratory behaviors from 72 unique elephants (including 28 individuals with 2 years of data); the 24 excluded elephants did not have a full calendar year of GPS fixes. We estimated annual displacement distance for the 28 elephants with two or more years of GPS fixes. We estimated 1254 monthly home ranges, movement distances, activity ratios and exploratory behaviors from 96 individuals (average per individual = 18; min = 3; max = 33). At least two successive months of data are required to estimate monthly displacement, thus the sample size for the site fidelity dataset reduced to 1198 observations (average per individual = 18; min = 2; max = 32).

### Covariates

We constructed rasters of environmental factors as follows: ‘annual temperature seasonality’ (the standard deviation in monthly temperature) and ‘total annual rainfall’ were obtained from the WorldClim database (http://www.worldclim.org/); mean daily rainfall for each month (October 2015-December 2018) was produced by averaging across interpolated daily rainfall Climate Hazards Group InfraRed Precipitation (CHIRPS) data; and the monthly normalized difference vegetation index (NDVI), consisting of 16-day MODIS composite imagery averaged across 2000 – 2018, was extracted from Google Earth Engine. Where cells lacked average NDVI data (typically due to cloud cover), they were assigned the NDVI score from the previous month. We averaged all monthly NDVI scores to derive the annual NDVI score for each raster cell. The anthropogenic disturbance factor, referred to as the Human Footprint Index (HFI), represents the Global Human Footprint Dataset of the Last of the Wild Project, Version 2 (2005)^46^. This layer is derived from nine data layers reflecting three key elements: human population (population density); human land use and infrastructure (built-up areas, nighttime lights, land use/land cover); and human access (coastlines, roads, railroads, navigable rivers). We extracted the mean raster value of each covariate within the home range of each individual (defined above) for each time step (monthly and yearly).

### Statistical modelling

We used a hierarchical modelling approach to (i) determine the key drivers of elephant movement behaviors in univariate models while controlling for individual, regional and temporal non-independence of the data, and (ii) assess the among-individual structure of behavioral (co)variation using multivariate models. All models were fit using the bayesian ‘MCMCglmm’ package^47^ within the R statistical environment V3.6.1^48^. Each behavioral response term was scaled to standard deviation units prior to analysis, facilitating comparisons of effect sizes across different traits in univariate models and assisting model convergence and interpretation in multivariate models. The continuous observed predictors were also standardized^49,50^. We consider fixed effects to be statistically significant if the 95% credible interval (95% CI) does not cross zero and discuss them in terms of their relative effect size. We ran all models for 850,000 iterations with a 50,000 burn-in and a thinning interval of 400, using the ‘coda’ package to determine the effective sample sizes^51^. All models fitted assumed (multivariate) Gaussian error structures, and we visually assessed residuals to verify this was reasonable (after data transformation in the case of home range size). We also visually assessed posterior distributions for stable model convergence and lack of autocorrelation, and checked that models were robust to different prior specifications on the random effects and that multiple runs converged to similar results. Individual-level repeatability in each behavioral trait and corresponding credible intervals were estimated using the formulation in^52^. We consider there to be support for non-negligible variance components and repeatability when the 95% CIs are distant from zero (variances cannot be negative, so their CIs cannot cross zero).

We ran separate univariate models for each behavioral trait at both the annual and monthly scales. To account for non-independence of repeated measures of individuals, we included individual identification (n = 96) and region (n = 7; defined as the protected area where elephants were originally collared) as random intercept terms. Monthly models also included ‘month’ as a random intercept to account for seasonal variation in movement behaviors. Given that correlation between candidate explanatory variables was low at both annual (mean *r* = 0.20; min = 0.04; max = 0.48) and monthly scales (mean *r* = 0.07; min = 0.03; max = 0.13), we constructed a global model with all candidate covariates as additive terms (sex, NDVI, human footprint index, temperature seasonality and rainfall) for each behavioral trait. We used the default inverse-gamma prior for residual variation (R: V = 1, v = 0.002) and an uninformative, parameter-expanded prior for each random effect (V = 1, nu = 1, αV = 625). Conditional and marginal R^2^ was calculated for each model following^52^ and presented in Supplementary Table 3.

To determine the among-individual relationships between different behaviors, we fit multi-response hierarchical models that enable us to partition multivariate behavioral (co)variation at multiple levels^53^. Because annual data have repeated measures for a small number of individuals, we performed this analysis on the monthly data only and included only those traits with significant among-individual variance as estimated in the univariate models. We fit all covariates as fixed effects on each response trait separately. We also fit unstructured covariance matrices at the individual, region, month, and residual (within-individual) levels, with priors on the variances as described for the univariate models above. We calculated correlations from the among-individual (co)variances as *r*_I(x,y)_ = COV_I(x,y)_/√(V_I(x)_ × V_I(y)_), and used the posterior distribution of derived correlations to estimate 95% CIs. Finally, we subjected the among-individual covariance matrix to eigen decomposition to assess whether a single major axis could explain most of the multivariate among-individual behavioral (co)variation^54^. We also used eigen decomposition of posterior draws to estimate 95% CIs on the trait loadings of these principal components.

## Results

### Distance moved

**Figure 2:**
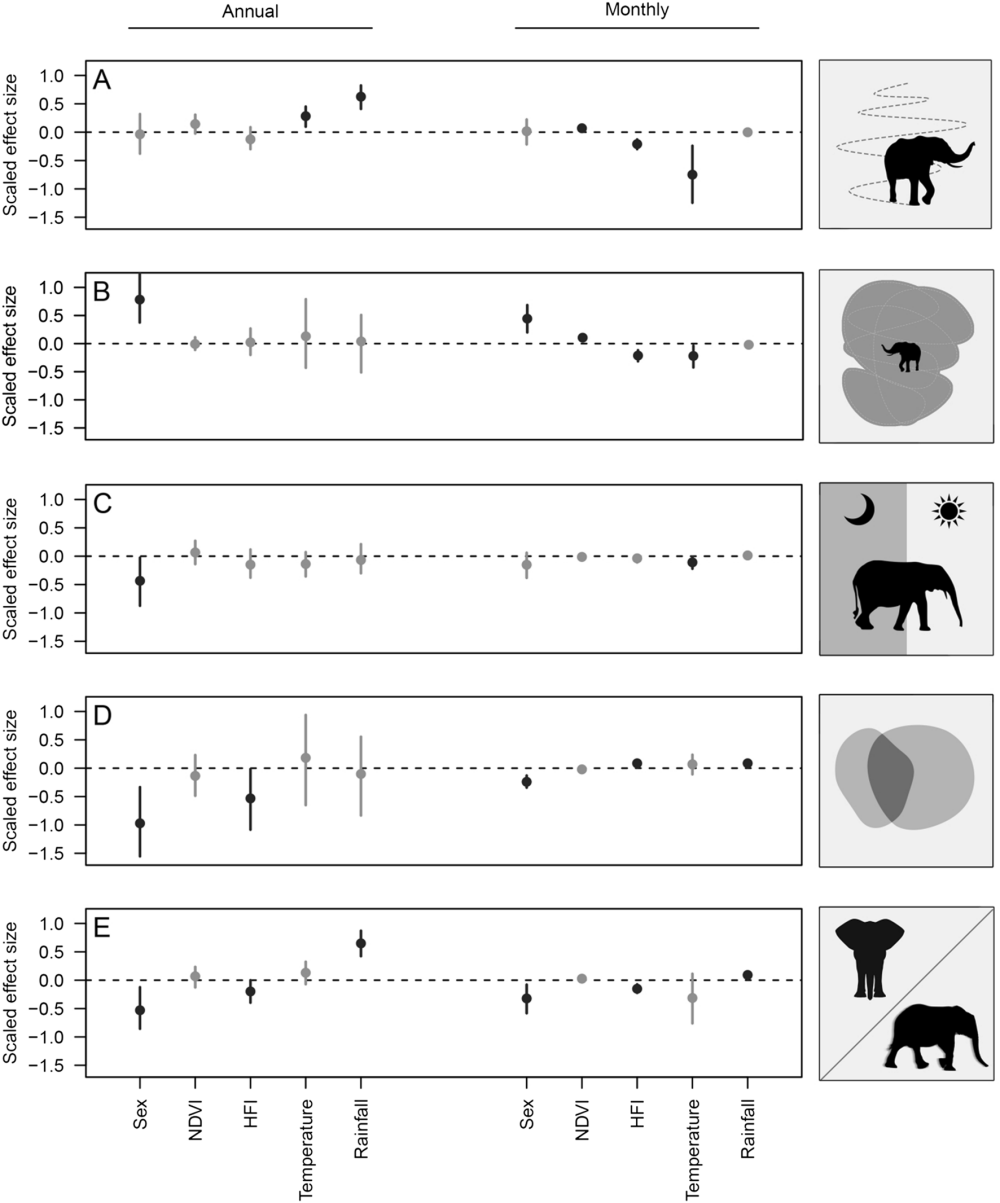
Relative effect size estimates (points) and 95% credible intervals (lines) for the predictors of elephant movement behavior (**A** = distance moved; **B** = home range size; **C ** = diurnality; **D** = site fidelity; **E** = proportion exploratory behavior) at two different scales (annual and monthly). Where: HFI = Human footprint Index; NDVI = Normalized Difference Vegetation Index; Temperature = temperature seasonality; Rainfall = mean annual rainfall in the annual models and monthly average rainfall in the monthly models; black = credible intervals which do not overlap zero; grey = credible intervals which do overlap zero.

The mean forest elephant movement distance was 2463 km annually (min = 1420 km; max = 3436 km). There was support for annual precipitation and temperature seasonality influencing annual movement distance (Fig. 2A). Annual rainfall most strongly increased annual movement distance with an effect size double that of rainfall seasonality (Fig. 2A; Supplementary Table 2). On average, movement distance increased by 59% from low to high rainfall and by 18% from low to high temperature seasonality (Supplementary Fig. 2). At the monthly scale, there was support for NDVI, HFI, and temperature seasonality influencing movement distance (Fig. 2A). Of the covariates with support, temperature seasonality and human disturbance had the largest, negative effects, on average decreasing movement distance 56% from low to high seasonality and by 58% from low to high human disturbance. NDVI weakly increased movement distance by 23% from low to high productivity (Fig. 2A; Supplementary Table 2; Supplementary Fig. 3).

### Home range

Forest elephants had average annual home range sizes of 195 km^2^. Of all predictors, there was only support for sex-specific home range sizes (Fig. 2B). Males were predicted to have average home ranges 80 km^2^ larger than females (males = 238 km^2^, 95% CI 161–332; females = 135 km^2^, 95% CI 109–220; Fig. 2B; Supplementary Fig. 4). At the monthly scale, elephants had an average home range size of 67 km^2^. Sex most strongly predicted monthly home range size, with average male home ranges 16 km^2^ larger than female home ranges (males = 76 km^2^, 95% CI 65–89; females = 60 km^2^, 95% CI 53–68; Fig. 2B; Supplementary Fig. 5), followed by human disturbance (predicted to reduce home range size by 63% from low to high disturbance; Supplementary Fig. 5) and NDVI (predicted to increase home range size by 50% from low to high productivity) (Fig. 2B; Supplementary Fig. 5).

### Diurnality

Forest elephants had a weak tendency to move farther during daylight hours than night at the annual scale (mean annual diurnality = 0.064; min = -0.08; max = 0.30), with males tending to be less active during the day than females (Fig. 2C; Supplementary Fig. 6). Although the means were similar, there was greater variation in monthly than annual diurnality (mean monthly diurnality = 0.076; min = -0.41; max = 0.30). Only temperature seasonality influenced monthly diurnality, with increasing temperature variation shifting activity towards nocturnality (Fig. 2C; Supplementary Fig. 7).

### Site fidelity

Annual home ranges overlapped by 85% from the first to the second year (min = 0.48%; max = 0.98%), suggesting strong inter-annual site fidelity. Males showed weaker site fidelity than females (79% vs. 90%) and fidelity decreased with increasing HFI (Fig. 2D; Supplementary Table 2; Supplementary Fig. 8). At the monthly scale, home range overlap declined to 61% (min = 0%; max = 100%). At the monthly scale, sex had the largest relative effect size, with males showing lower site fidelity than females, whereas HFI and rainfall both had weaker positive effects on fidelity (Fig. 2D; Supplementary Fig. 9). However, the results of the site fidelity models must be interpreted with caution due to their low effective sample sizes (suggesting poor model convergence, Supplementary Table 2).

### Exploratory behavior

Annually, forest elephants spent 56% of their time in exploratory states (min = 23%; max = 82%). Rainfall most strongly predicted exploratory behavior, with higher rainfall increasing exploration, followed by sex, with males exploring less than females. The effects of rainfall and sex were similar in magnitude (Fig. 2E; Supplementary Table 2; Supplementary Fig. 10). HFI also had a weak, negative effect on exploratory behavior. At the monthly scale, sex, HFI and rainfall influenced exploratory behavior (Fig. 2E). As in the annual model, males engaged in less exploratory behavior than females and increasing HFI was correlated with decreased exploration. Exploratory behavior also increased with increasing monthly rainfall (Fig. 2E; Supplementary Table 2; Supplementary Fig. 11).

### Variance components

Inspection of variance components from annual models suggested that all behaviors are highly repeatable (Table 1). However, only a small proportion (28/72) of individuals had annual repeated measurements, thus these results are likely driven by an inability to separate among- from within-individual variance. At the monthly scale, all 96 individuals had multiple observations, and we found low to moderate repeatability for 4 of the 5 behaviors (Table 1). There was no individual repeatability in site fidelity, with 95% of the phenotypic variation (after accounting for fixed effects) apportioned to the residual variance component, suggesting that this trait is highly variable within individuals. At both monthly and annual scales, and across all traits, ‘region’ typically explained little to no variation (Supplementary Table 3).

**Table 1:** Individual repeatability from the best supported univariate models of each movement behavior at each spatial scale (annual and monthly).

Behavior	Annual		Monthly	
	R_adj_	95% CI	R_adj_	95% CI
Movement distance	0.695	0.491–0.835	0.095	0.016–0.177
Home range size	0.916	0.598–0.994	0.269	0.189–0.350
Site fidelity	–	–	0.000	–
Diurnality	0.847	0.733–0.934	0.213	0.120–0.312
Exploratory movement	0.658	0.431–0.843	0.203	0.061–0.329

Where: individual repeatability (*R*_*adj*_) is the proportion of variance attributable to among-individual differences after controlling for the fixed effects; 95% CI is the 95% credible interval calculated from the posterior distribution around the individual repeatability estimate. For conditional and marginal R^2^ from each univariate model see Supplementary Table 3.

### Between-individual behavioral correlations

**Figure 3:**
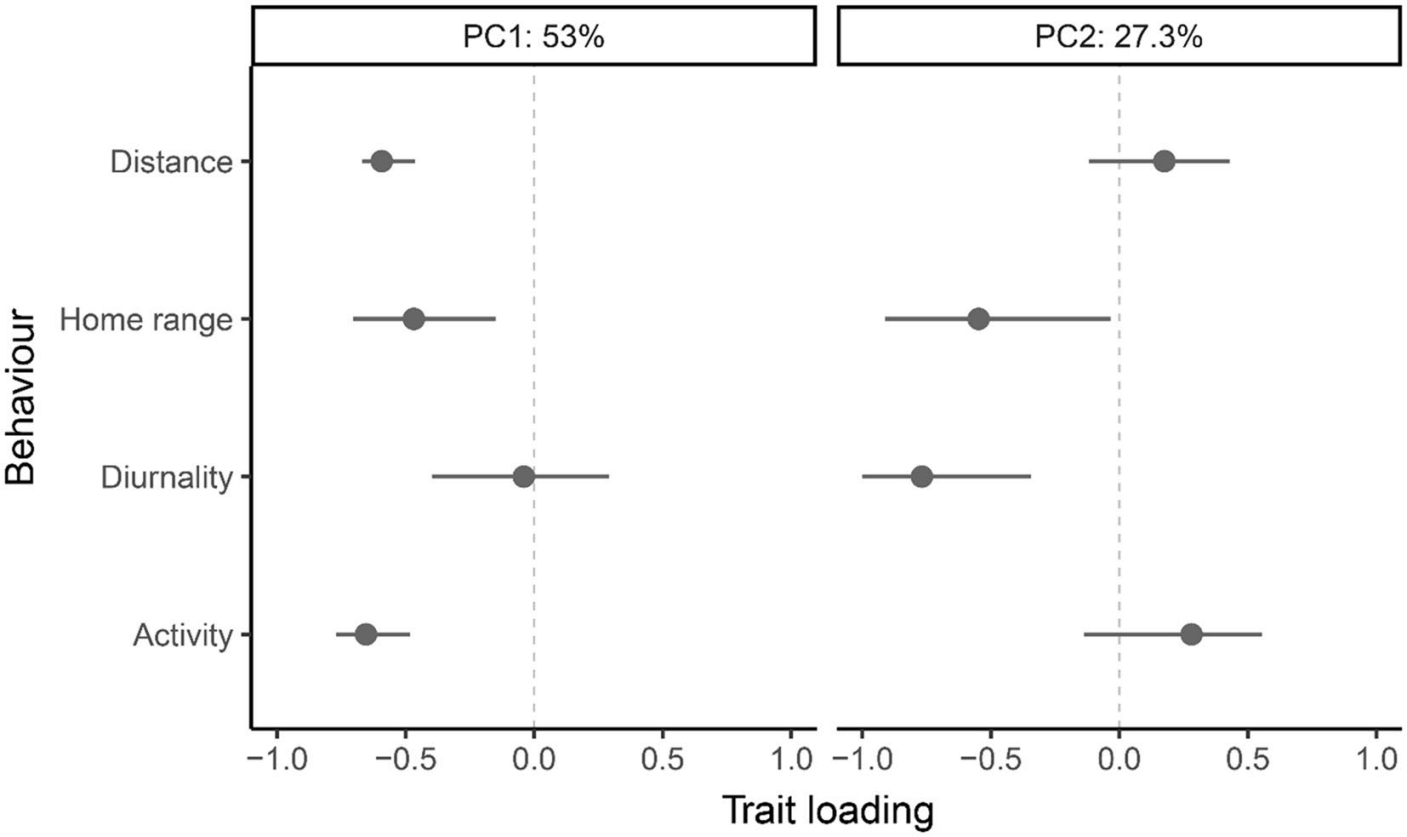
Trait loadings on the first two eigenvectors (PC1, left; PC2, right) from the among-individual covariance matrix of four movement behaviors at the monthly scale. Points represent estimates, and lines represent 95% credible intervals calculated from posterior distributions. The arithmetic sign of the loading denotes the grouping of behaviors (i.e., PC1 represents an axis where one extreme features individuals that travel long distances, show high rates of activity, and have large home ranges and the other extreme features elephants that travel short distances, show low activity, and have small home ranges).

Examination of the among-individual correlations between traits (Table 2, upper-diagonals) showed several significant pairwise relationships, namely between distance, home range and exploratory behavior. Our eigen analysis identified a large major axis of individual behavioral variation in forest elephants, explaining almost double the variation as the second axis (Fig. 3). The first major axis loaded heavily on distance, home range and exploratory behavior (in the same direction). The second axis explained more than a quarter of the total among-individual variation, suggesting an association between home range and diurnality among individual elephants.

**Table 2:** Among-individual variance–covariance matrix for movement behaviors at the monthly level.

	Distance	Home range	Diurnality	Exploratory behaviour
Distance	*0.20 (0.13, 0.28)*	**0.41 (0.22, 0.5)**	− 0.03 (− 0.46, 0.17)	**0.87 (0.83, 0.88)**
Home range	**0.09 (0.03, 0.16)**	*0.25 (0.17, 0.36)*	0.21 (− 0.09, 0.34)	**0.32 (0.06, 0.43)**
Diurnality	− 0.01 (− 0.06, 0.05)	0.05 (− 0.01, 0.11)	*0.21 (0.13, 0.29)*	− 0.05 (− 0.49, 0.16)
Exploratory behavior	**0.20 (0.12, 0.27)**	**0.08 (0.01, 0.15)**	− 0.01 (− 0.07, 0.05)	*0.25 (0.16, 0.34)*

Among-individual variances (V_I,_ analogous to repeatability over the full range of behavioral measurements) are given on the italicised diagonal (top-left to bottom-right), with among-individual between-trait covariances (COV_I_) below and the corresponding correlations (*r*_I_) above. 95% credible intervals are provided in parentheses and bolded numbers highlight between-trait covariances and correlations  that do not overlap 0.

## Discussion

Using the largest dataset of forest elephant movements to date, we assessed the factors influencing elephant movement behavior at a landscape scale in Gabon. Several of the key drivers of elephant movement are scale dependent—the factors influencing annual movement differ from those that influence monthly movement. We also found evidence for consistent differences in monthly movement behaviors among individual elephants and correlations between behaviors that suggest the existence of behavioral syndromes. Our results highlight the challenges facing practitioners trying to understand fine-scale elephant movement behaviors and suggest future avenues to mitigate human-wildlife conflict. We discuss each of the drivers of elephant movement below and their implications for conservation and management.

Sex was a key driver of elephant movement behavior in four of five behavioral traits. Males generally possess larger home ranges, show lower site fidelity, are slightly more nocturnal, and spend less time in exploratory movements than females. Such sex-based differences likely reflect differences in energy budgets: adult males are typically larger than females and may need to spend more time foraging (less time in directed movements) over a wider area than females^55^. That said, as observed in savannah elephants, there is considerable overlap in home range size between the sexes (Supplementary Fig. 4), potentially reflecting behavioral changes in males associated with their age or reproductive status^55^. The slightly higher degree of nocturnality in males is consistent with stronger poaching pressure on them for ivory^25^, potentially causing males to avoid diurnal anthropogenic disturbance through shifted activity patterns. Longer movement distance, larger home range size and weaker site fidelity of male elephants than females potentially bring them into greater contact with poachers. Consistent with this, by the end of 2019, three male elephants in our study population had been killed by poachers. Incorporating spatially explicit poaching information (threat, extent) may help explain the magnitude of sex differences observed.

Anthropogenic disturbance, based on the human footprint index, influenced four of five movement behaviors: distance moved, home range size, diurnality and proportion of directed movements. In agreement with a multi-taxonomic assessment of the effect of anthropogenic disturbance on animal movement^56^, increasing anthropogenic disturbance generally reduced elephant movement (shorter movement distances, smaller home range sizes and fewer exploratory movements). These results suggest that forest elephants act consistently with the 'landscape of fear' hypothesis, altering their behavior in response to human activity. Ironically, these differences could also signal reliance on humans: elephants living near humans might raid plantations, heightening human-wildlife conflict. Both mechanisms would cause forest elephants to use smaller home ranges than in protected landscapes^57^. However, the direction of the effect of anthropogenic disturbance on diurnality changed at different time scales: diurnality decreased with higher anthropogenic disturbance at the annual scale and increased at the monthly scale, perhaps capturing behavioural differences between elephants which are consistently exposed to human disturbance versus those which experience it transiently. Forest elephants might modify their behavior due to direct interactions with humans and anthropogenic features (e.g., roads or farmland) or indirect responses to changing habitat structure (e.g., changing species composition of logged/disturbed forests). Consideration of the factors influencing elephant movement at smaller time scales (e.g., diel) will be required to examine the mechanisms driving these patterns.

Forage availability has been highlighted as a key factor influencing forest elephant habitat selection^58^, but we found little support for effects of NDVI on most movement behaviors. When NDVI was retained in our models, it had very small positive effects on home range and movement distance relative to sex and anthropogenic disturbance. NDVI might be a more reliable indicator of habitat selection rather than movement behaviors within habitats, as in^34^. Forest elephants have remarkably broad diets, consuming the leaves and bark of hundreds of plant species^15,59,60^. Therefore, NDVI, a coarse remotely-sensed vegetative density, is unlikely to reflect the true diversity, abundance and quality of forage available at any given time-step, particularly at large spatial scales with high habitat heterogeneity. NDVI also does not capture the availability of ripe fruit, a key factor mediating forest elephant movement behavior^60^, which may explain why home ranges appear to increase as NDVI increases at the monthly scale. Thus, adapting approaches which facilitate fine temporal and spatial-scale fruit availability, through the use of drones^61^ or intensive in-the-field follows^60^, may improve the explanatory power of resource availability in movement models.

Despite inhabiting rainforests, forest elephants are often limited by water availability^34,60^. Consistent with earlier studies, this work suggests increased rainfall leads to longer movement distances and more directed movements, suggesting that individuals can take prolonged excursions away from perennial water sources when rainwater is abundant and ephemeral streams and ponds have water. The positive relationship between high temperature seasonality, movement distance and nocturnality might also reflect water-stress if elephants reduce the distance they travel and/or shift to nocturnal activity when temperatures are high. The links between elephant movement behavior and rainfall/temperature seasonality highlight the need to explicitly consider water availability for the designation of elephant corridors: elephants may be reluctant to use habitat too far from perennial water sources or do so only in the wet season. Although the effects of climate change in central Africa are understudied, some projections predict lower future rainfall^62^, which might strengthen the effects of water limitation on elephant distribution and movement. Long term reductions in rainfall have also been linked to dramatic reductions in fruit availability and corresponding declines in forest elephant body condition^63^, which will likely act synergistically with water availability to influence forest elephant movement behaviors^60^.

In addition to environmental and anthropogenic drivers of elephant movement, we identified consistent significant differences among individuals in their movement behavior: individuals behave differently from one another. Crucially, these differences cannot be attributed to ecological context, as the ‘region’ (the national parks or general geographic area to which an elephant belonged), explained very little variation across both scales and all traits (Supplementary Table 2). Our multivariate analysis also provided evidence for consistent individual differences in the relationships between movement behaviors, consistent with the concepts of ‘behavioral syndromes’ or ‘personalities’. A large proportion of this (co)variation loaded on a single axis that varies from individuals with smaller home ranges, shorter movement distances and less exploratory behavior to those with larger ranges, longer movement distances and who are more exploratory. Therefore, elephants seem to exhibit personalities along an ‘idler’ to ‘explorer’ axis. Such information could be used to identify “problem individuals”^12^ whose suite of behavioral traits could bring them into frequent human-wildlife conflict or poaching risk. Clearly, anthropogenic, climatic and resource availability metrics are insufficient to capture the variation both between and within individuals. This is not surprising given the complex social and environmental cues to which elephants are known to respond. The significant variation between individuals suggests the possibility for heritable and/or learned variation in movement behaviors^64^. Movement behaviors are likely under selection due to human-wildlife conflict, and so there is the potential for an evolutionary response in movement behaviors with further downstream effects. For example, if far-ranging males are more frequently poached than more stationary males, poaching could select for elephants with higher site fidelity, changing the population genetic structure and potentially limiting their role in long distance seed dispersal^15,59^.

Elephant movement behavior is more consistent at annual than monthly time frames: elephants behave similarly from year to year. Other species similarly manifest scale-dependent behavioral repeatability^65^. However, the high repeatability in annual behaviors in our study may be driven in part by low sample size (n = 28). Elephants are very long-lived, thus assessing the long-term repeatability of movement behaviors requires a multi-year dataset. The strong heterogeneity in month-to-month behavior highlights the difficulty in predicting elephant movement behavior at finer temporal scales. Interestingly, anthropogenic disturbance influenced 4 of 5 behaviors at the monthly scale and only 2 of 5 behaviors at the annual scale. The influence of anthropogenic disturbance on movement behaviors may be further magnified at diel scales when elephants face decisions like whether to approach or escape areas with high anthropogenic activity. Furthermore, the effect directions of some drivers of elephant movement were generally, but not always, consistent across temporal scales, underscoring the danger of extrapolating inference beyond the temporal scale of analysis.

Our analyses represent the first standardized, national-scale synthesis of forest elephant movement. Both extrinsic environmental and anthropogenic drivers and intrinsic individual variation strongly affected movement behaviors of forest elephants, highlighting the complexity of modelling their movement. If the aim is to create models that can predict human-wildlife conflict with high spatial and temporal accuracy, then future models should include additional intrinsic attributes (e.g., age, body condition), finer extrinsic information (e.g., fruit and forage availability, poaching pressure) and finer scale movement data (e.g., 15-min intervals). In this study, we demonstrate evidence for generalities in the drivers of elephant movement behavior, of which sex and rainfall had the most consistent effects: compared to females, males had large home range size, low site fidelity, high nocturnality and more frequent encamped behaviors and higher rainfall increased exploratory movements and annual home range size. Other drivers, such as anthropogenic disturbance, had scale specific effects that differed between annual and monthly timeframes. Moreover, we identified movement-related behavioral syndromes: individuals who move more typically have larger home ranges and engage in more exploratory behavior (‘explorers’). We also found marked, repeatable, among-individual variation in movement traits, suggesting that elephants have personalities and that no two elephants behave the same. This variation among individuals could complicate the development of general strategies for conservation if elephants differentially respond to management (e.g., McDougall et al., 2006) but it also accentuates the importance of conserving such a wide-ranging, intelligent and socially-complex species.

## Supplementary Information

Supplementary Information.
