# Supplementary material for: African forest elephant movements depend on time scale and individual behavior
Source: Sci Rep. 2021 Jun 16;11:12634. doi: 10.1038/s41598-021-91627-z (PMC8208977; doi:10.1038/s41598-021-91627-z)
Supplement: Supplementary file 1 — Supplementary Information. [file 41598_2021_91627_MOESM1_ESM.docx]

**Supplementary material for**

**African forest elephant movements depend on time scale and individual behavior**

Christopher Beirne^a^, Thomas M. Houslay^b^, Peter Morkel^c^, Connie J. Clark^a^, Mike Fay^d^, Joseph Okouyi^d^, Lee J.T. White^d,e,f^, and John R. Poulsen^a†^

^a^Nicholas School of the Environment, Duke University, PO Box 90328, Durham, NC, 27708

^b^Department of Zoology, University of Cambridge, Cambridge, CB2 3EJ, UK

^c^Independent Researcher, Karasburg, Namibia

^d^Agence Nationale des Parcs Nationaux, Batterie IV, BP. 20379, Libreville, Gabon

^e^Institut de Recherche en Ecologie Tropicale, BP. 13354, Libreville, Gabon

^f^African Forest Ecology Group, School of Natural Sciences, University of Stirling, Stirling, UK

**^†^Corresponding author:** john.poulsen@duke.edu

**Supplementary Table 1**

**Supplementary Table 1:** Elephants included in the study and their known metadata

| **Name** | **Sex** | **Region of initial collaring** | **Estimated age range** |
| --- | --- | --- | --- |
| Abu | Male | Loango | NA |
| Amelia | Female | Ivindo | NA |
| Angelia | Female | Moukalaba Doudou | NA |
| Annabelle_ww | Female | Wonga Wongue | NA |
| Beti | Male | Nyonie | NA |
| Boniface | Male | Loango | NA |
| BraBrou | Male | Wonga Wongue | NA |
| David | Male | Wonga Wongue | NA |
| Dedi | Female | Moukalaba Doudou | NA |
| Doudou | Female | Moukalaba Doudou | NA |
| Dumbo | Male | Loango | NA |
| Ekare | Male | Loango | NA |
| Estell | Female | Loango | NA |
| Freddy | Male | Nyonie | NA |
| Izouwa | Female | Loango | NA |
| Janice | Female | Ivindo | NA |
| Jolie | Male | Moukalaba Doudou | NA |
| Junior | Male | Loango | NA |
| Kalie | Male | Loango | NA |
| Kandji | Female | Loango | NA |
| Kengue | Male | Wonga Wongue | NA |
| Keva | Female | Loango | NA |
| Kigali | Male | Wonga Wongue | NA |
| Koumba | Male | Moukalaba Doudou | NA |
| Larouille | Male | Ivindo | NA |
| Lisa | Female | Wonga Wongue | NA |
| Malaika | Female | Wonga Wongue | NA |
| Mambo | Male | Wonga Wongue | NA |
| Marijo | Female | Ivindo | NA |
| Matamba | Female | Moukalaba Doudou | NA |
| Mba | Male | Wonga Wongue | NA |
| Mboumba | Male | Wonga Wongue | NA |
| Megane | Female | Moukalaba Doudou | NA |
| Moukalaba | Female | Moukalaba Doudou | NA |
| Mpemba | Female | Loango | NA |
| Nana | Female | Wonga Wongue | NA |
| Ndeka | Female | Wonga Wongue | NA |
| Ngele | Female | Nyonie | NA |
| Nkoghe | Male | Ivindo | NA |
| Nongo | Female | Wonga Wongue | NA |
| Nzamba | Male | Ivindo | NA |
| Nze | Male | Wonga Wongue | NA |
| Onero | Male | Loango | NA |
| Orembo | Male | Loango | NA |
| Penelope | Female | Moukalaba Doudou | NA |
| Ponogo | Female | Loango | NA |
| Program | Female | Moukalaba Doudou | NA |
| Rosa | Female | Wonga Wongue | NA |
| Rose_Loango | Female | Loango | NA |
| Stam | Female | Wonga Wongue | NA |
| Stephanie | Female | Nyonie | NA |
| Ta_a | Male | Ivindo | NA |
| Tokende | Female | Loango | NA |
| Tonda | Female | Loango | NA |
| Tonnere | Male | Wonga Wongue | NA |
| Wongo | Male | Wonga Wongue | NA |
| Chouchou | Female | Ivindo | 25-40 |
| Mabekwe | Female | Mwagne | 40+ |
| Ketelyne | Female | Ivindo | 25-40 |
| Tito | Male | Ivindo | 20-25 |
| Janette | Female | Ivindo | 20-25 |
| Patrice | Male | Ivindo | 25-40 |
| Louaye | Male | Mwagne | 50+ |
| Jovis | Male | Ivindo | NA |
| Odile | Female | Ivindo | 25-40 |
| Kate | Female | Mwagne | 40+ |
| Gracia | Female | Minkebe | 25-40 |
| Synthia | Female | Mwagne | 25-40 |
| Syndie | Female | Mwagne | 25-40 |
| Charlotte | Female | Minkebe | 20-25 |
| Yeleh | Female | Mwagne | 25-40 |
| Harvey | Male | Ivindo | 20-25 |
| Nouna | Female | Minkebe | 40+ |
| Zara | Female | Mwagne | 25-40 |
| Dr_Flore | Female | Mwagne | 40+ |
| Madiba | Male | Mwagne | 40+ |
| Cheri | Female | Minkebe | 40+ |
| Lodjie | Female | Mwagne | 25-40 |
| Jessica | Female | Minkebe | 25-40 |
| Ada | Female | Minkebe | 25-40 |
| Marie | Female | Minkebe | 40+ |
| Neky | Female | Minkebe | 40+ |
| Toto | Female | Minkebe | 25-40 |
| Souvenir | Female | Minkebe | 40+ |
| Harry | Male | Minkebe | 20-25 |
| Vian | Female | Minkebe | 25-40 |
| Andrea | Female | Loango | NA |
| Mouyeket | Female | Ivindo | 20-25 |
| Vicky | Female | Ivindo | 25-40 |
| Emeline | Female | Ivindo | 25-40 |
| CEB | Female | Ivindo | 20-25 |
| Loubi | Female | Ivindo | 20-25 |
| Doutsona | Female | Ivindo | 40+ |
| Lea | Female | Ivindo | 40+ |
| Virginie | Female | Ivindo | 50+ |
| Lassio | Female | Ivindo | 25-40 |

**Supplementary Figure 1**


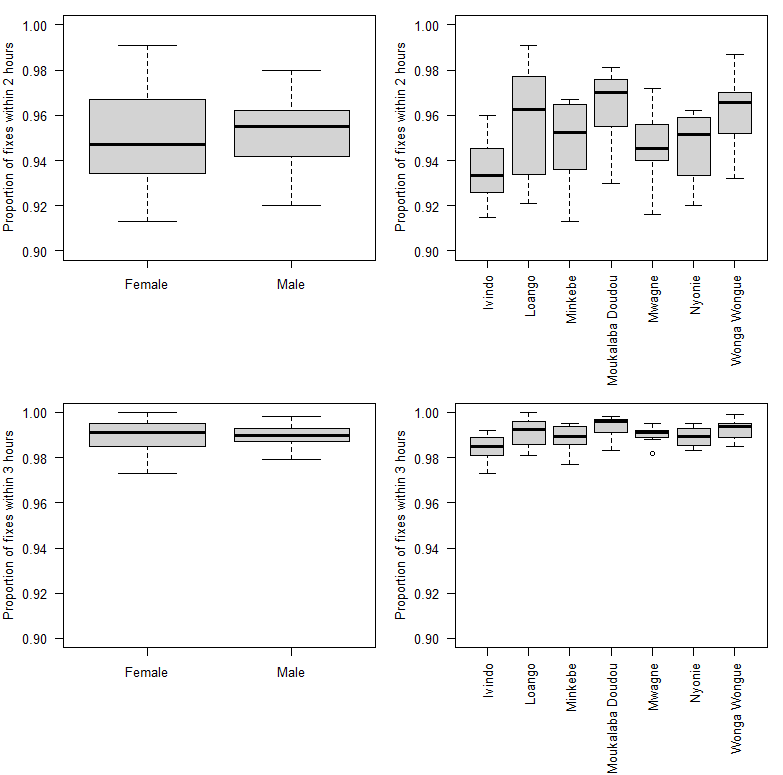


**Supplementary Figure 1: Boxplots of the proportion of fixes occurring within two hours (upper) and three hours (lower) of the previous fix by sex (left) and region (right).** Where: thick black lines represent the media value; boxes represent the interquartile range; and whiskers denote the extreme values.

**Supplementary Table 2**

**Supplementary Table 2:** Full output for standardized univariate models. Where: L95/U95 = the lower and upper 95% credible intervals calculated from the posterior distribution; ESS = Effective Sample Size for each parameter.

| **Trait** | **Scale** | **Covariate** | **Mean** | **L95** | **U95** | **ESS** |
| --- | --- | --- | --- | --- | --- | --- |
| Home range | Annual | Intercept | -0.3967 | -1.0827 | 0.2879 | 1808 |
| Home range | Annual | Sex | 0.7824 | 0.3748 | 1.2624 | 2000 |
| Home range | Annual | NDVI | -0.0074 | -0.1130 | 0.1130 | 1880 |
| Home range | Annual | HFI | 0.0224 | -0.2013 | 0.2681 | 1775 |
| Home range | Annual | Temp | 0.1304 | -0.4301 | 0.7898 | 1497 |
| Home range | Annual | Rain | 0.0395 | -0.5122 | 0.5089 | 1824 |
| Diurnality | Annual | Intercept | 0.1676 | -0.1514 | 0.4685 | 2000 |
| Diurnality | Annual | Sex | -0.4522 | -0.8884 | -0.0123 | 2000 |
| Diurnality | Annual | NDVI | 0.0594 | -0.1386 | 0.2764 | 2000 |
| Diurnality | Annual | HFI | -0.1499 | -0.4076 | 0.0701 | 2000 |
| Diurnality | Annual | Temp | -0.1317 | -0.3607 | 0.0824 | 2138 |
| Diurnality | Annual | Rain | -0.0652 | -0.3301 | 0.2145 | 2000 |
| Movement distance | Annual | Intercept | -0.0176 | -0.2667 | 0.2270 | 2000 |
| Movement distance | Annual | Sex | -0.0384 | -0.3707 | 0.3231 | 2000 |
| Movement distance | Annual | NDVI | 0.1427 | -0.0429 | 0.2985 | 2000 |
| Movement distance | Annual | HFI | -0.1319 | -0.3213 | 0.0536 | 2000 |
| Movement distance | Annual | Temp | 0.2831 | 0.0891 | 0.4814 | 2000 |
| Movement distance | Annual | Rain | 0.6287 | 0.3862 | 0.8410 | 2000 |
| Fidelity | Annual | Intercept | 0.3704 | -0.5137 | 1.2211 | 2000 |
| Fidelity | Annual | Sex | -0.9498 | -1.6266 | -0.3853 | 2000 |
| Fidelity | Annual | NDVI | -0.1343 | -0.4904 | 0.2568 | 2000 |
| Fidelity | Annual | HFI | -0.5321 | -1.0960 | 0.0039 | 2000 |
| Fidelity | Annual | Temp | 0.1752 | -0.7559 | 0.9749 | 2000 |
| Fidelity | Annual | Rain | -0.1309 | -0.9036 | 0.7218 | 1771 |
| Exploratory movement | Annual | Intercept | 0.2230 | -0.0322 | 0.4667 | 2231 |
| Exploratory movement | Annual | Sex | -0.5100 | -0.8779 | -0.1449 | 2000 |
| Exploratory movement | Annual | NDVI | 0.0758 | -0.1216 | 0.2649 | 2000 |
| Exploratory movement | Annual | HFI | -0.1937 | -0.3957 | 0.0012 | 1843 |
| Exploratory movement | Annual | Temp | 0.1327 | -0.0433 | 0.3355 | 2000 |
| Exploratory movement | Annual | Rain | 0.6504 | 0.4282 | 0.8628 | 1695 |
| Home range | Monthly | Intercept | -0.1906 | -0.4504 | 0.0645 | 2182 |
| Home range | Monthly | Sex | 0.4438 | 0.1994 | 0.6853 | 2000 |
| Home range | Monthly | NDVI | 0.1055 | 0.0440 | 0.1626 | 2000 |
| Home range | Monthly | HFI | -0.2126 | -0.3152 | -0.1167 | 2000 |
| Home range | Monthly | Temp | -0.2200 | -0.4243 | -0.0371 | 1736 |
| Home range | Monthly | Rain | -0.0205 | -0.0827 | 0.0384 | 1456 |
| Diurnality | Monthly | Intercept | 0.0011 | -0.4351 | 0.4301 | 2000 |
| Diurnality | Monthly | Sex | -0.1426 | -0.3570 | 0.0764 | 2009 |
| Diurnality | Monthly | NDVI | -0.0132 | -0.0580 | 0.0332 | 2000 |
| Diurnality | Monthly | HFI | -0.0386 | -0.1087 | 0.0277 | 2000 |
| Diurnality | Monthly | Temp | -0.1051 | -0.2088 | -0.0079 | 2100 |
| Diurnality | Monthly | Rain | 0.0152 | -0.0501 | 0.0792 | 2000 |
| Movement distance | Monthly | Intercept | -0.4436 | -1.7163 | 0.9325 | 2000 |
| Movement distance | Monthly | Sex | 0.0123 | -0.2251 | 0.2465 | 2000 |
| Movement distance | Monthly | NDVI | 0.0685 | 0.0228 | 0.1135 | 1869 |
| Movement distance | Monthly | HFI | -0.2073 | -0.2842 | -0.1324 | 2000 |
| Movement distance | Monthly | Temp | -0.7557 | -1.2524 | -0.2782 | 2000 |
| Movement distance | Monthly | Rain | -0.0021 | -0.0677 | 0.0620 | 2222 |
| Fidelity | Monthly | Intercept | 0.0627 | -0.1755 | 0.2895 | 1634 |
| Fidelity | Monthly | Sex | -0.2396 | -0.3396 | -0.1344 | 2000 |
| Fidelity | Monthly | NDVI | -0.0205 | -0.0702 | 0.0265 | 2000 |
| Fidelity | Monthly | HFI | 0.0864 | 0.0293 | 0.1493 | 2000 |
| Fidelity | Monthly | Temp | 0.0666 | -0.0922 | 0.2211 | 1865 |
| Fidelity | Monthly | Rain | 0.0842 | 0.0348 | 0.1331 | 1670 |
| Exploratory movement | Monthly | Intercept | -0.1639 | -0.8807 | 0.6197 | 2000 |
| Exploratory movement | Monthly | Sex | -0.3202 | -0.5772 | -0.0709 | 2000 |
| Exploratory movement | Monthly | NDVI | 0.0259 | -0.0195 | 0.0702 | 2000 |
| Exploratory movement | Monthly | HFI | -0.1532 | -0.2229 | -0.0708 | 2000 |
| Exploratory movement | Monthly | Temp | -0.3180 | -0.7411 | 0.0957 | 2000 |
| Exploratory movement | Monthly | Rain | 0.0920 | 0.0339 | 0.1565 | 2000 |

**Supplementary Table 3**

**Supplementary Table 3:** Full output for standardized univariate models. Where: L95/U95 = the lower and upper 95% credible intervals calculated from the posterior distribution; ESS = Effective Sample Size for each parameter.

| **Trait** | **Scale** | **Level** | **Mean** | **L95** | **U95** |
| --- | --- | --- | --- | --- | --- |
| Home range | Annual | Total | 0.988 | 0.979 | 0.997 |
| Home range | Annual | Individual | 0.741 | 0.248 | 0.990 |
| Home range | Annual | Region | 0.243 | 0.000 | 0.745 |
| Home range | Annual | Fixed | 0.208 | 0.025 | 0.401 |
| Diurnality | Annual | Total | 0.880 | 0.794 | 0.940 |
| Diurnality | Annual | Individual | 0.849 | 0.746 | 0.938 |
| Diurnality | Annual | Region | 0.009 | 0.000 | 0.044 |
| Diurnality | Annual | Fixed | 0.151 | 0.037 | 0.283 |
| Movement distance | Annual | Total | 0.835 | 0.725 | 0.922 |
| Movement distance | Annual | Individual | 0.680 | 0.462 | 0.859 |
| Movement distance | Annual | Region | 0.021 | 0.000 | 0.137 |
| Movement distance | Annual | Fixed | 0.440 | 0.278 | 0.595 |
| Fidelity | Annual | Total | 1.159 | 0.085 | 2.640 |
| Fidelity | Annual | Individual | 0.104 | 0.000 | 0.873 |
| Fidelity | Annual | Region | 0.896 | 0.127 | 1.000 |
| Fidelity | Annual | Fixed | 0.464 | 0.049 | 0.727 |
| Exploratory movement | Annual | Total | 0.758 | 0.418 | 0.910 |
| Exploratory movement | Annual | Individual | 0.603 | 0.000 | 0.796 |
| Exploratory movement | Annual | Region | 0.007 | 0.000 | 0.021 |
| Exploratory movement | Annual | Fixed | 0.374 | 0.222 | 0.517 |
| Home range | Monthly | Total | 0.455 | 0.304 | 0.699 |
| Home range | Monthly | Individual | 0.273 | 0.190 | 0.376 |
| Home range | Monthly | Region | 0.052 | 0.000 | 0.205 |
| Home range | Monthly | Fixed | 0.124 | 0.052 | 0.195 |
| Diurnality | Monthly | Total | 0.654 | 0.534 | 0.775 |
| Diurnality | Monthly | Individual | 0.213 | 0.116 | 0.303 |
| Diurnality | Monthly | Region | 0.002 | 0.000 | 0.007 |
| Diurnality | Monthly | Fixed | 0.024 | 0.003 | 0.049 |
| Movement distance | Monthly | Total | 0.869 | 0.756 | 0.972 |
| Movement distance | Monthly | Individual | 0.096 | 0.019 | 0.184 |
| Movement distance | Monthly | Region | 0.635 | 0.322 | 0.940 |
| Movement distance | Monthly | Fixed | 0.181 | 0.017 | 0.373 |
| Fidelity | Monthly | Total | 0.099 | 0.032 | 0.212 |
| Fidelity | Monthly | Individual | 0.001 | 0.000 | 0.003 |
| Fidelity | Monthly | Region | 0.063 | 0.005 | 0.176 |
| Fidelity | Monthly | Fixed | 0.037 | 0.013 | 0.062 |
| Exploratory movement | Monthly | Total | 0.772 | 0.639 | 0.923 |
| Exploratory movement | Monthly | Individual | 0.202 | 0.064 | 0.329 |
| Exploratory movement | Monthly | Region | 0.355 | 0.047 | 0.720 |
| Exploratory movement | Monthly | Fixed | 0.110 | 0.011 | 0.245 |

**Supplementary Figure 2: Annual distance moved**


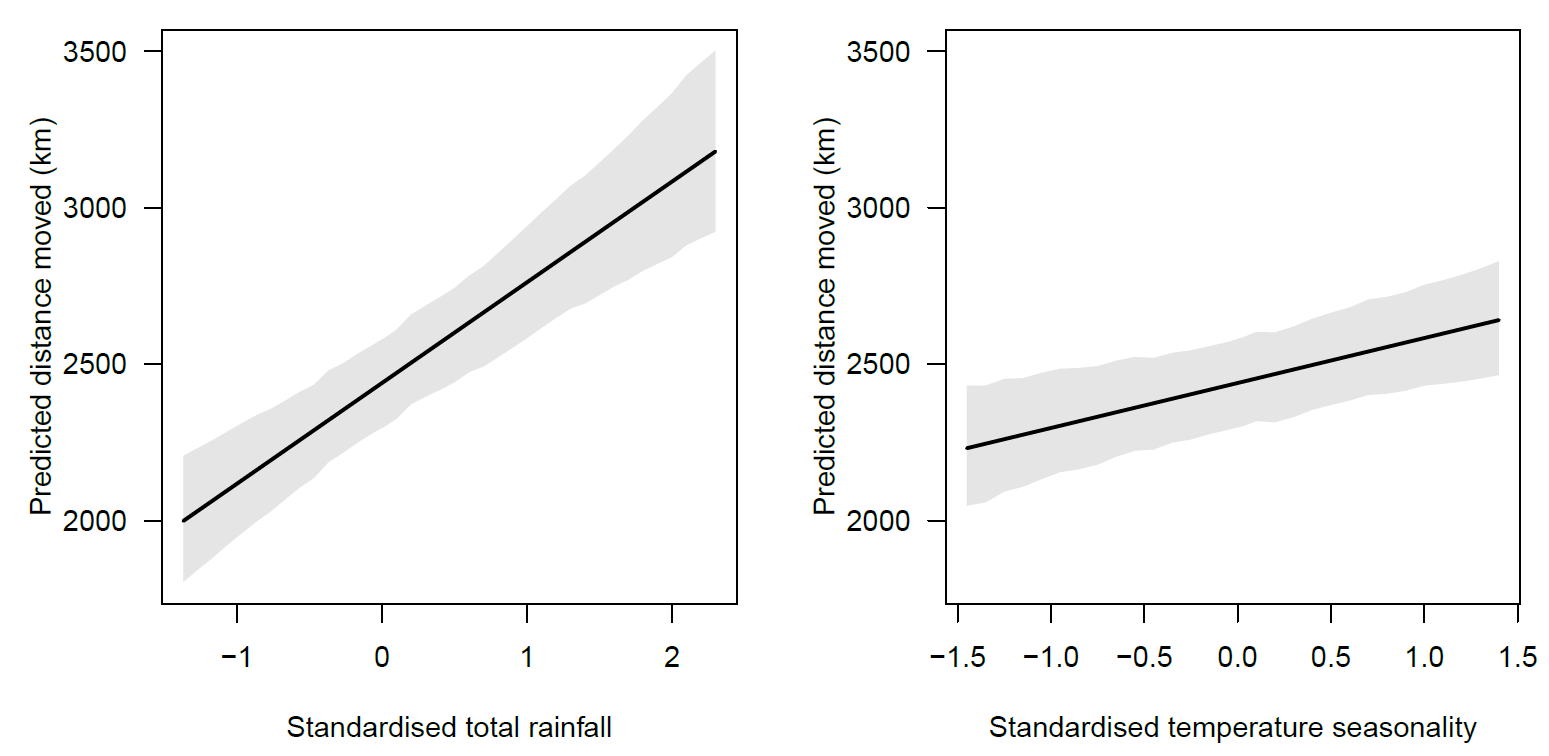


**Supplementary Figure 2:** Model predictions on the original scale for each of the predictors of elephant behavior with statistical support (credible intervals do not overlap zero). Where: black line = mean effect; grey polygon = 95% credible interval.

**Supplementary Figure 3: Monthly distance moved**
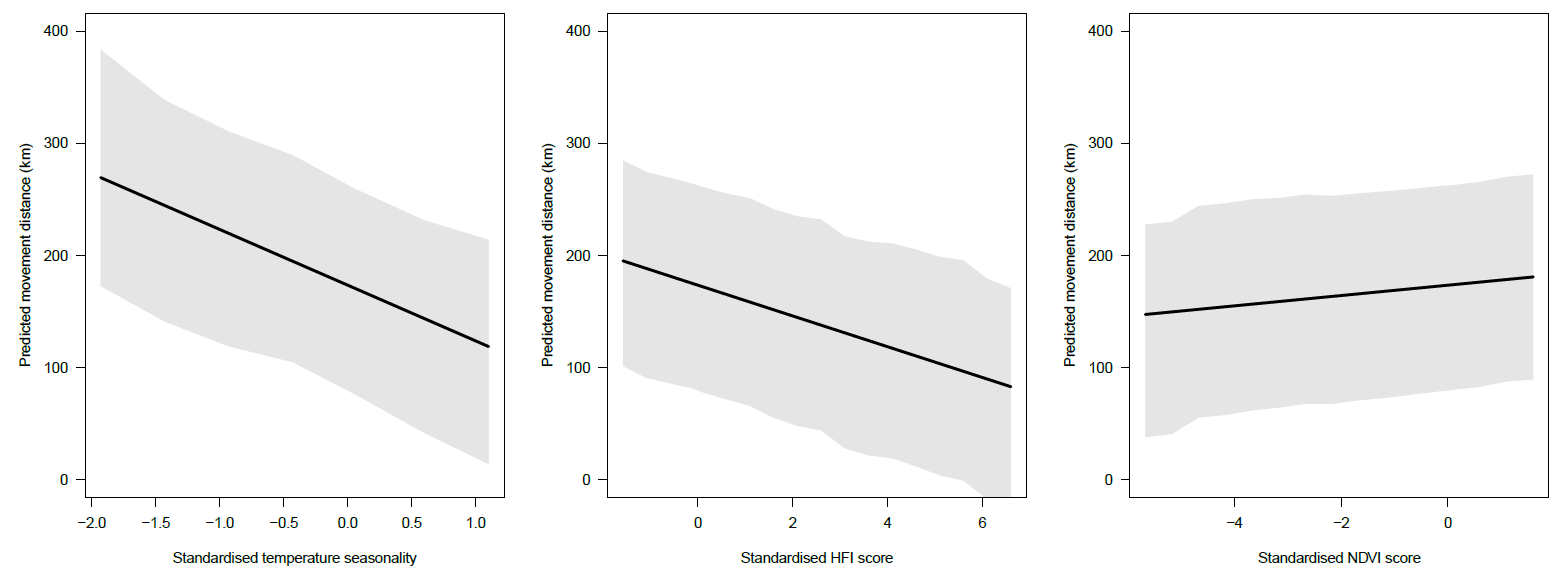


**Supplementary Figure 3:** Model predictions on the original scale for each of the predictors of elephant behavior with statistical support (credible intervals do not overlap zero). Where: black line = mean effect; grey polygon = 95% credible interval.

**Supplementary Figure 4: Annual home range**


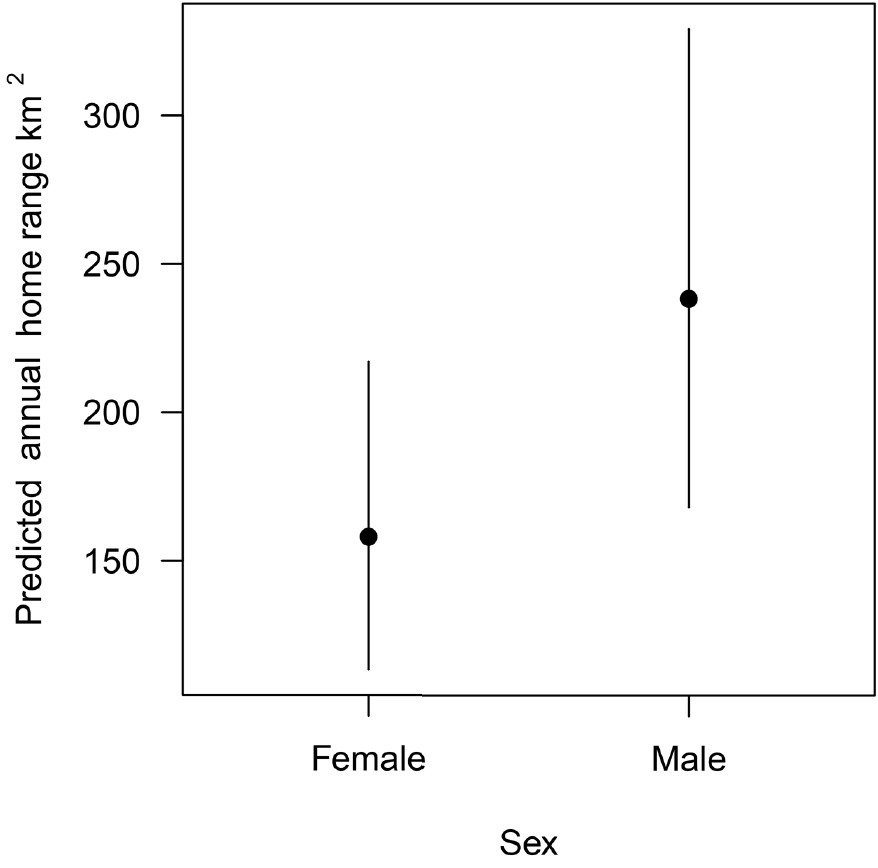


**Supplementary Figure 4:** Model predictions on the original scale for each of the predictors of elephant behavior with statistical support (credible intervals do not overlap zero). Where: black line = mean effect; grey polygon = 95% credible interval.

**Supplementary Figure 5: Monthly home range**


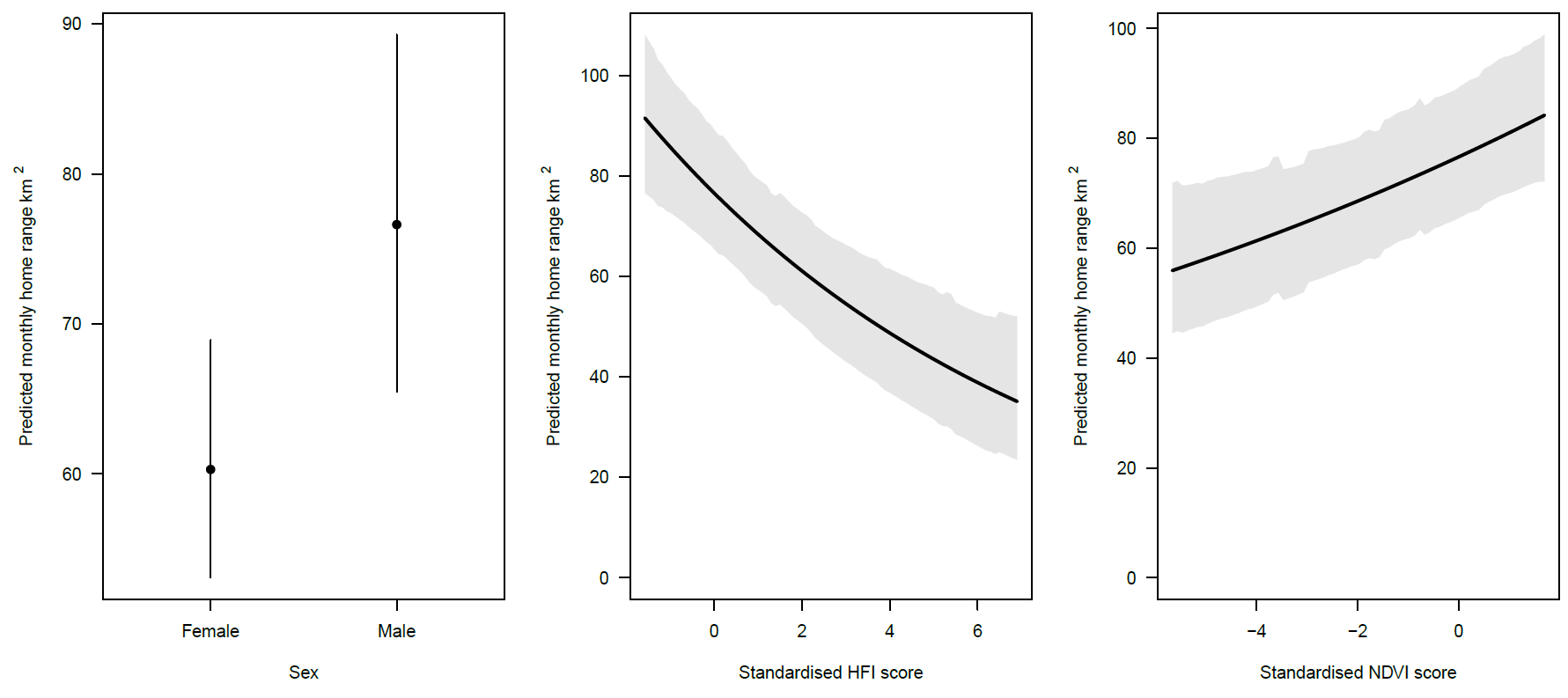


**Supplementary Figure 5:** Model predictions on the original scale for each of the predictors of elephant behavior with statistical support (credible intervals do not overlap zero). Where: black line = mean effect; grey polygon = 95% credible interval.

**Supplementary Figure 6: Annual Diurnality**


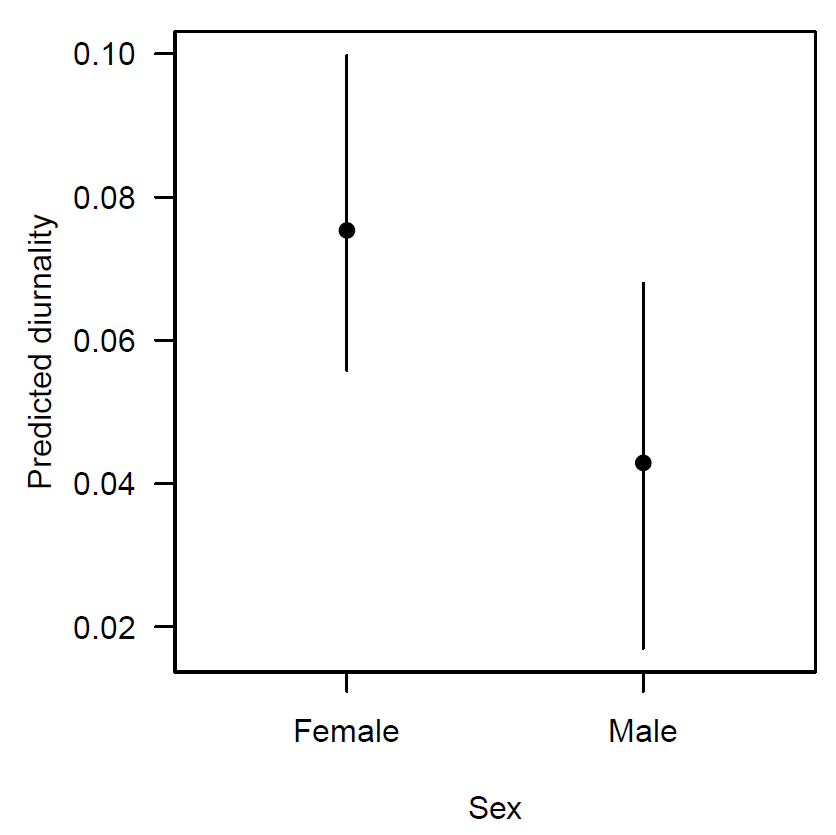


**Supplementary Figure 6:** Model predictions on the original scale for each of the predictors of elephant behavior with statistical support (credible intervals do not overlap zero). Where: black line = mean effect; grey polygon = 95% credible interval.

**Supplementary Figure 7: Monthly Diurnality**


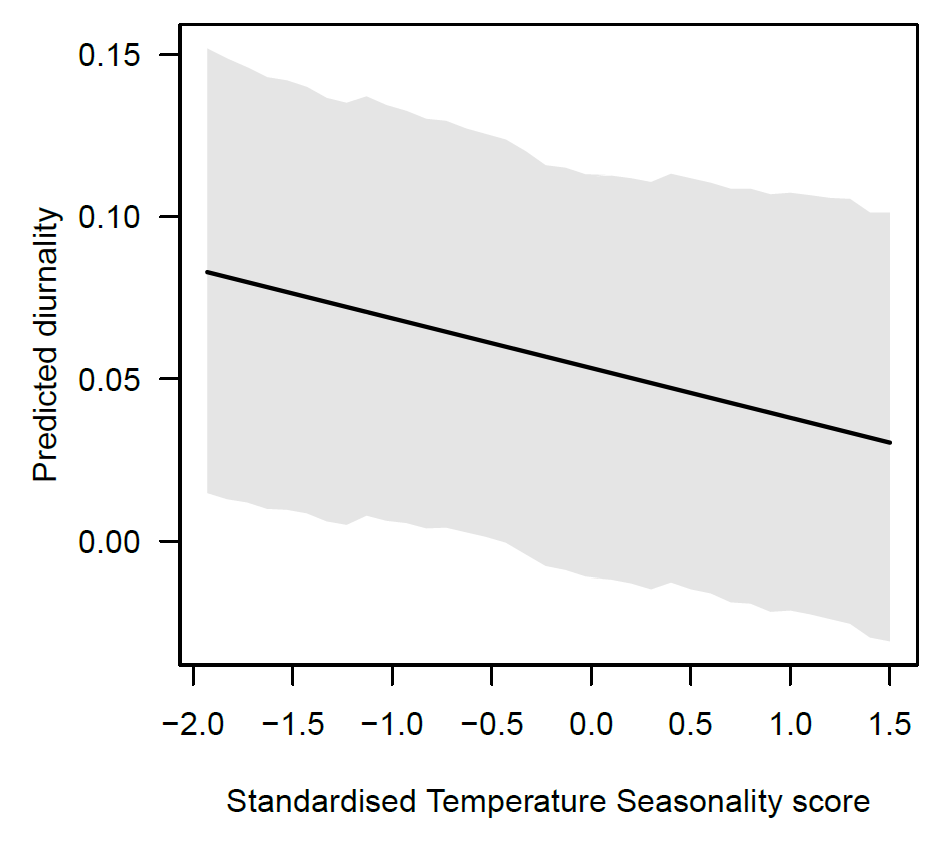


**Supplementary Figure 7:** Model predictions on the original scale for each of the predictors of elephant behavior with statistical support (credible intervals do not overlap zero). Where: black line = mean effect; grey polygon = 95% credible interval.

**Supplementary Figure 8: Annual site fidelity**


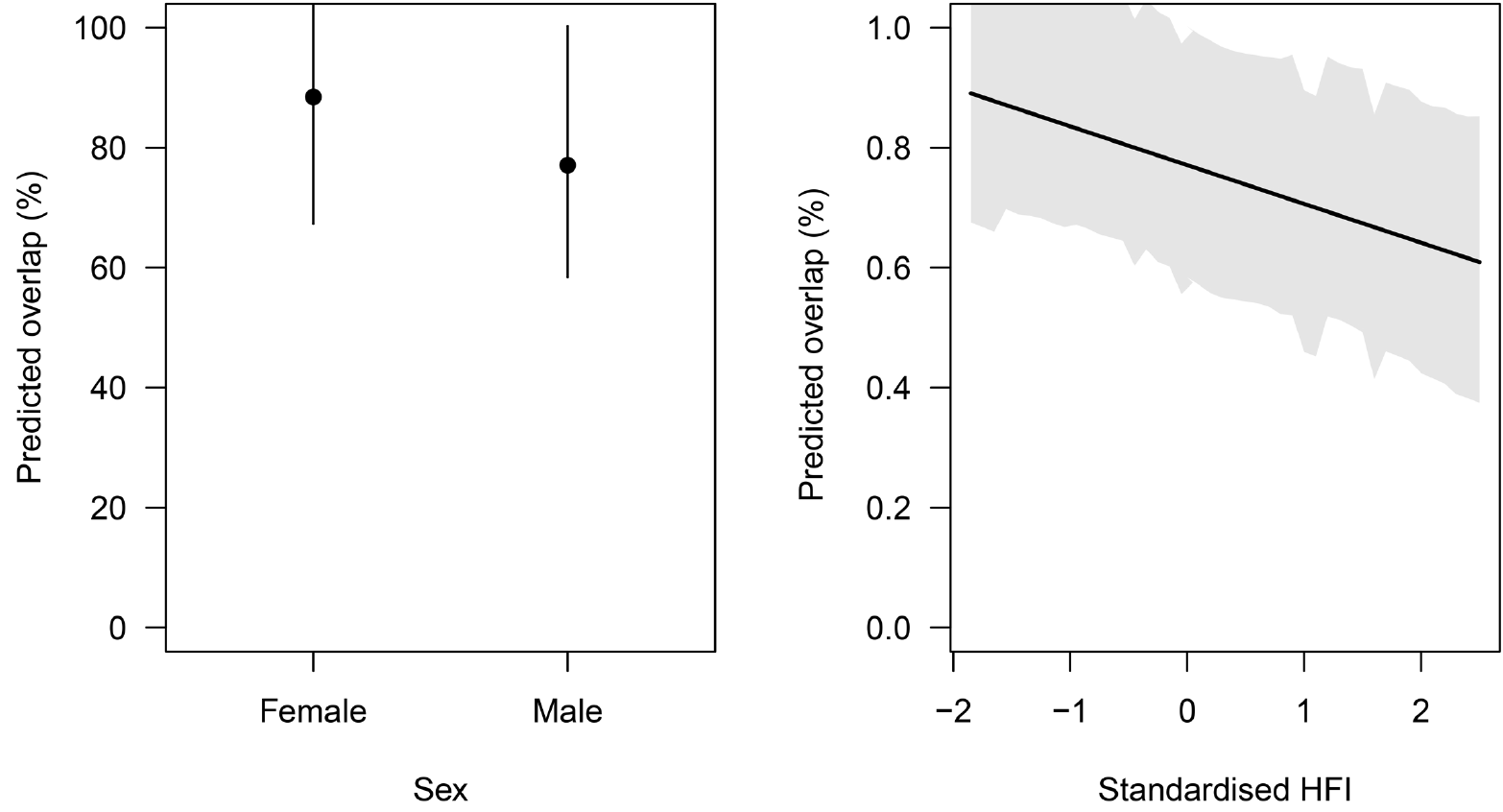
 **Supplementary Figure 8:** Model predictions on the original scale for each of the predictors of elephant behavior with statistical support (credible intervals do not overlap zero). Where: black line = mean effect; grey polygon = 95% credible interval.

**Supplementary Figure 9: Monthly site fidelity**


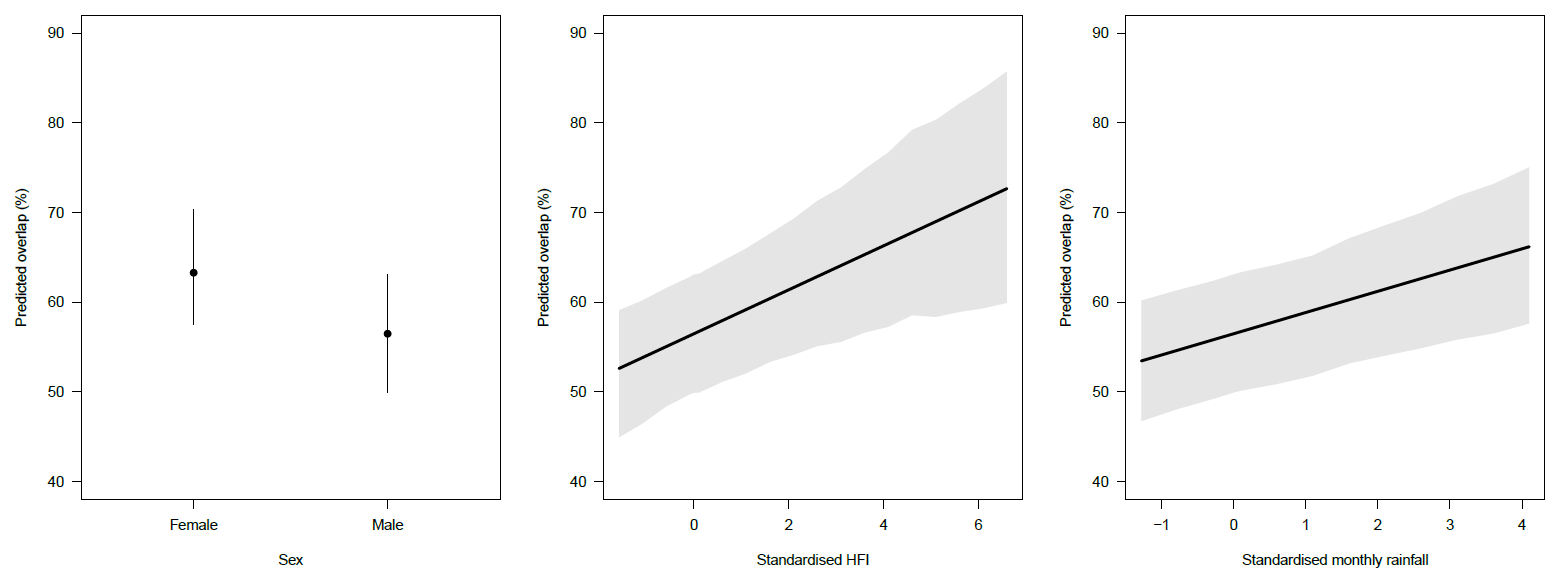


**Supplementary Figure 9:** Model predictions on the original scale for each of the predictors of elephant behavior with statistical support (credible intervals do not overlap zero). Where: black line = mean effect; grey polygon = 95% credible interval.

**Supplementary Figure 10: Annual exploratory behavior**


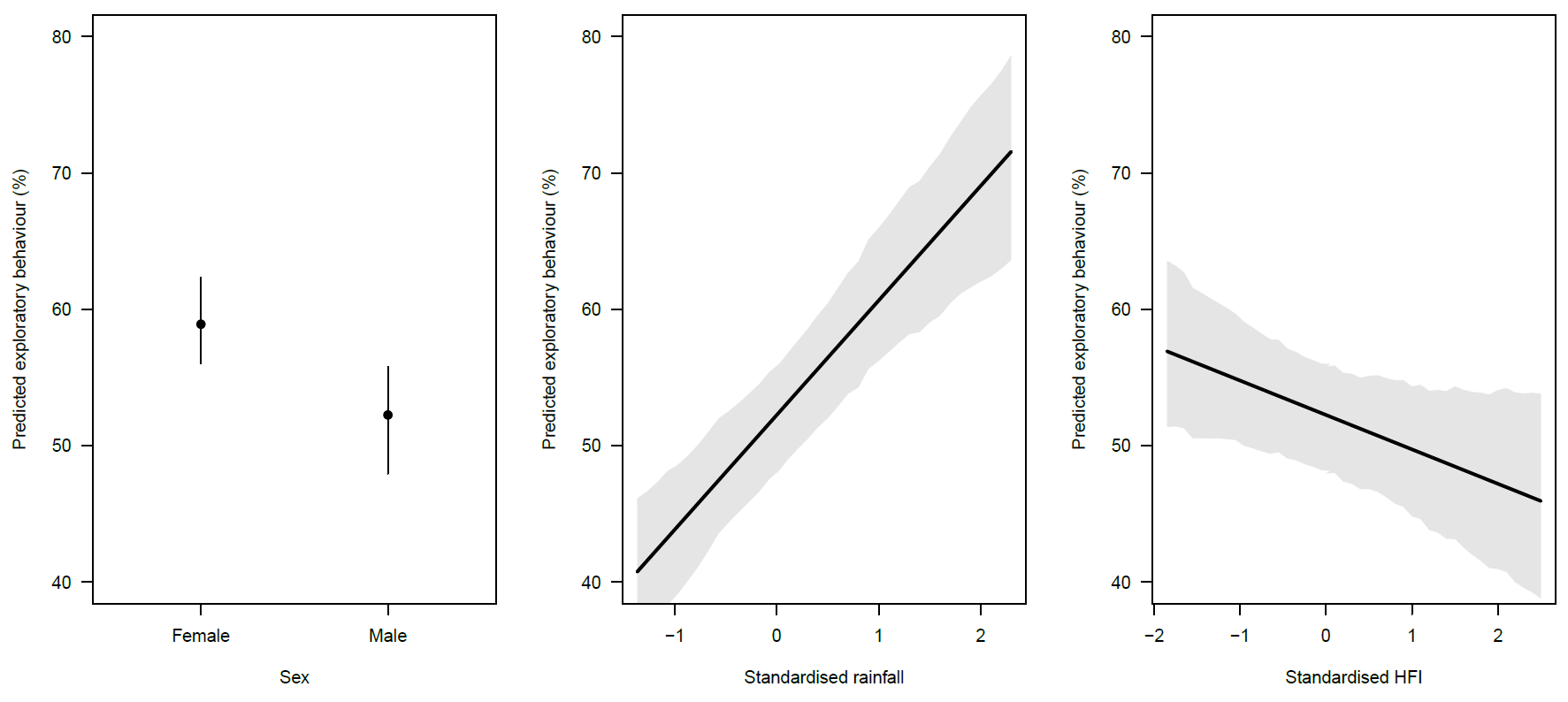


**Supplementary Figure 10:** Model predictions on the original scale for each of the predictors of elephant behavior with statistical support (credible intervals do not overlap zero). Where: black line = mean effect; grey polygon = 95% credible interval.

**Supplementary Figure 11: Monthly exploratory behavior**


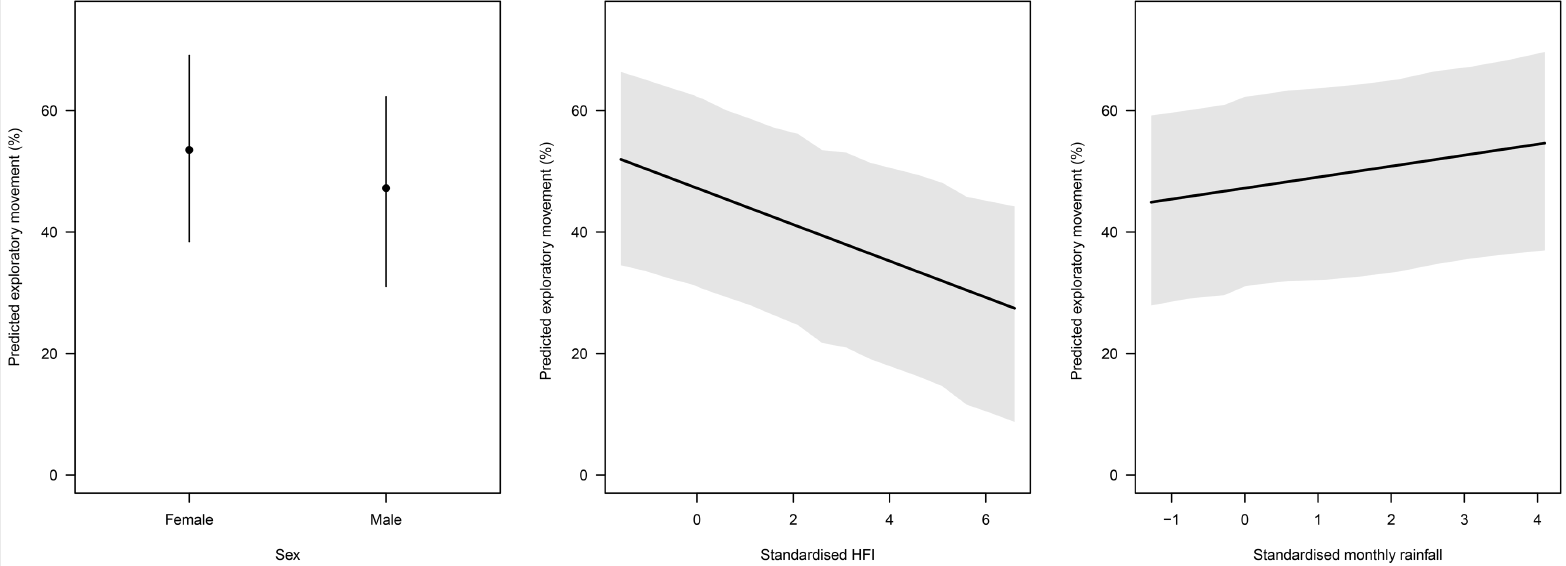


**Supplementary Figure 11:** Model predictions on the original scale for each of the predictors of elephant behavior with statistical support (credible intervals do not overlap zero). Where: black line = mean effect; grey polygon = 95% credible interval.
